# 

**Published:** 1868

**Authors:** 


### CONTENTS.

### Number III.

### ORIGINAL COMMUNICATIONS.

PAGE.

ARTICLE I.—Ceanothus Americanus. By D. L. Phares, M. D., Newtonia, Miss..... 129

ARTICLE II.—Diagnosis of Idiopathic Fevers. By F. M. Brantly, M. D., Rocky Mount, Ga....... 132

### SELECTIONS.

ARTICLE III.—The Contagiousness of the General Symptoms of Syphilis. By Freeman J. Bumstead, M. D , Prof. of Venereal Diseases at the College of Physicians and Surgeons, New York....... 135

ARTICLE IV.—Indigenous Remedies of the Southern States. By Joseph Jones, M. D., Prof. of Physiology and Pathology in the Medical Department of the University of Nashville, Tenn....... 136

ARTICLE IV.—Physiological View of the Nervous System, and its Disorders. By E. A. Kunkler, M. D., Placerville, Cal....... 157

ARTICLE VI.—Experimental Medicine, &c. By Dr. L. Ranvier, Paris. Translated by Walter Hay, Chicago....... 162

ARTICLE VII.—On the Import of Symptoms. By Samuel Henry Dickson, M. D., LL. D., Prof. etc., Jefferson Medical College, Philadelphia....... 173

### EDITORIAL AND MISCELLANEOUS.

Medical Colleges....... 186

Account of the Four-Legged Child, J. Myrtle Corban....... 189

